# Is there a trade-off between peak performance and performance breadth across temperatures for aerobic scope in teleost fishes?

**DOI:** 10.1098/rsbl.2016.0191

**Published:** 2016-09

**Authors:** Julie J. H. Nati, Jan Lindström, Lewis G. Halsey, Shaun S. Killen

**Affiliations:** 1 Institute of Biodiversity, Animal Health and Comparative Medicine, University of Glasgow, Graham Kerr Building, Glasgow G12 8QQ, UK; 2 Department of Life Sciences, University of Roehampton, Holybourne Avenue, London SW15 4JD, UK

**Keywords:** ecophysiology, metabolic rate, locomotion, thermal performance, environmental change

## Abstract

The physiology and behaviour of ectotherms are strongly influenced by environmental temperature. A general hypothesis is that for performance traits, such as those related to growth, metabolism or locomotion, species face a trade-off between being a thermal specialist or a thermal generalist, implying a negative correlation between peak performance and performance breadth across a range of temperatures. Focusing on teleost fishes, we performed a phylogenetically informed comparative analysis of the relationship between performance peak and breadth for aerobic scope (AS), which represents whole-animal capacity available to carry out simultaneous oxygen-demanding processes (e.g. growth, locomotion, reproduction) above maintenance. Literature data for 28 species indicate that peak aerobic capacity is not linked to thermal performance breadth and that other physiological factors affecting thermal tolerance may prevent such a trade-off from emerging. The results therefore suggest that functional links between peak and thermal breadth for AS may not constrain evolutionary responses to environmental changes such as climate warming.

## 1. Introduction

**Figure 1.**
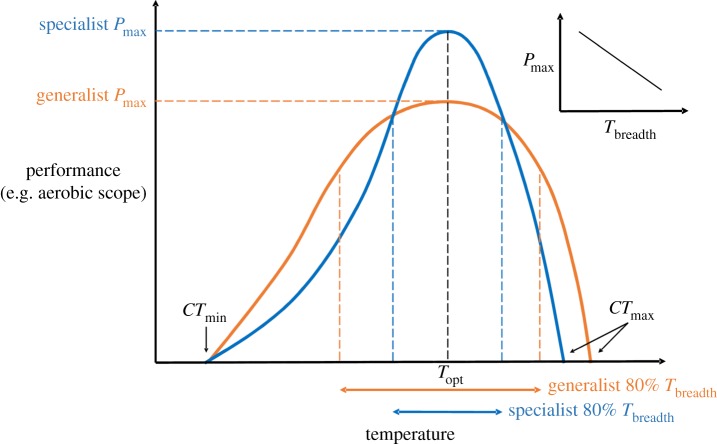
Theoretical thermal performance curves illustrating thermal specialists (higher peak performance, in blue) and thermal generalists (higher performance breadth, in orange). Specialists have a higher peak performance (*P*_max_) at their optimum temperature (*T*_opt_). Generalists have a lower *P*_max_ but a wider breadth of temperatures over which they perform normally (*T*_breadth_, here defined as the range of temperatures allowing 80% of *P*_max_). Critical minimum and maximum thermal limits (*CT*_min_ and *CT*_max_, respectively) occur where performance equals zero. Inset: predicted negative correlation between *P*_max_ and *T*_breadth_ if there is a trade-off between being a thermal specialist and a thermal generalist.

For ectotherms, performance traits related to growth, reproduction and locomotion are often depicted using thermal performance curves [1,2] that illustrate how a trait responds to variation in environmental temperature (figure 1). Evolutionary thermal adaptation [2] may result in thermal specialists or thermal generalists, performing better over a narrow versus a broad range of temperatures, respectively (figure 1; [3]). Thermal and biochemical constraints on enzyme structure and function and membrane fluidity suggest that adaptations for increased performance at one temperature may cause decreased performance at other temperatures, resulting in a trade-off between peak performance (*P*_max_) at a thermal optimum and thermal performance breadth (*T*_breadth_). Owing to these potential compromises, previous researchers have suggested that a ‘jack of all temperatures is a master of none’ [4].

While a trade-off between *P*_max_ and *T*_breadth_ is predicted by theory [3,5], several studies have documented that an increased performance capacity at one temperature does not necessarily lead to reduced performance at other temperatures [4,6–9]. Notably, however, most studies have examined the differences in performance among populations of the same species, with few attempts to examine whether a trade-off exists across species. Therefore, it remains unknown whether any trade-off between *P*_max_ and *T*_breadth_ generate interspecific constraints on thermal adaptation. Furthermore, most attempts to examine trade-offs between *P*_max_ and *T*_breadth_ have focused on isolated components of locomotory performance (e.g. maximum speed, endurance). This approach may, however, fail to detect broader-scale trade-offs in organismal functioning. Aerobic scope (AS), in contrast, is an integrative trait, representing whole-animal cardiovascular and respiratory capacity to provide oxygen above maintenance requirements, for aerobic activities including growth, locomotion and reproduction [10,11]. In ectotherms, AS generally increases with temperature until *T*_opt_ and then usually decreases with further warming ([12], but see [13] for exceptions), potentially providing a composite measure to examine thermal sensitivity of whole-animal aerobic performance. AS is also ecologically relevant and has been related to geographical distribution [14], the capacity to cope with environmental stressors [15] and competitive ability [16]. Additionally, species with a higher AS tend to be more active and athletic [17], presumably because increased locomotion requires a greater allocation of oxygen to skeletal muscles [12]. If there is indeed a trade-off between *P*_max_ and *T*_breadth_, then ectothermic species that experience selection for increased peak AS to facilitate foraging, predator avoidance or migration may conversely have a reduced *T*_breadth_ [12].

We investigated the relationship between *P*_max_ and *T*_breadth_ for AS across 28 species of teleost fish, to determine the extent to which adaptation for performance at a particular optimal temperature may impose constraints on performance across a range of temperatures. We focus on fishes because they are a diverse taxon that experiences shifts in thermal regimes over varying timescales. It has also been proposed that AS may influence the ability of fishes to respond to climate change [18].

## 2. Material and methods

Data for the AS of fish, calculated as the difference between standard and maximum metabolic rates (SMR and MMR), were extracted from the literature where they were available at three or more temperatures for a species within a single study (*n* = 28 species; electronic supplementary material, table S1). A Gaussian model was fitted to the relationship between AS and temperature for each species to produce a thermal performance curve for each species [19]. Peak AS (*P*_max_) was defined as the highest value for AS along the thermal performance curve. The optimum temperature (*T*_opt_) was the temperature corresponding to *P*_max_ [1,11]. We used one dataset per species; when more than one dataset was available, we used the dataset that measured AS at the highest number of temperatures. If multiple datasets were available for a species within a given study, then we used that which gave the highest value for *P*_max_. Performance breadth (*T*_breadth_) was the range of temperatures over which a species maintained at least 80% of peak AS [20]. As the 80% of *P*_max_ designation for *T*_breadth_ range is somewhat arbitrary, we also examined 60%, 70% and 90% thresholds for *T*_breadth_. The modelled values for *T*_opt_ and *P*_max_ versus the highest values for measurement temperature and AS, respectively, which were observed in each source study are given in electronic supplementary material, figure S1. Data for an additional 15 species were not included because either: (i) AS did not increase or decrease appreciably over the temperatures tested (i.e. the performance ‘curve’ was flat; two species); (ii) AS decreased with no obvious peak across temperatures tested in the study, perhaps, because the lowest temperature in the study was above *T*_opt_ (three cases); (iii) AS increased with no obvious peak across temperatures tested in the study, perhaps, because the highest temperature in the study was below *T*_opt_ (nine cases) or (iv) AS continued to increase with temperature until *CT*_max_ (one case).

Data were analysed using the phylogenetic generalized least-squares (PGLS) method applying a phylogeny generated from the comprehensive tree of life [21] (electronic supplementary material, figure S2 for more details on statistical analysis, including phylogenetic tree). Log *P*_max_ (mg O_2_ h^−1^) was the response variable with *T*_breadth_, *T*_opt_ and log body mass (g) as explanatory variables. Six studies measured AS in fish acutely exposed to each temperature; we therefore constructed a separate model using only studies in which fish were thermally acclimated (*n* = 20 species, electronic supplementary material, table S2). Model residuals were checked for normality and homogeneity of variance. The significance level of all tests was *α* = 0.05.

## 3. Results

**Figure 2.**
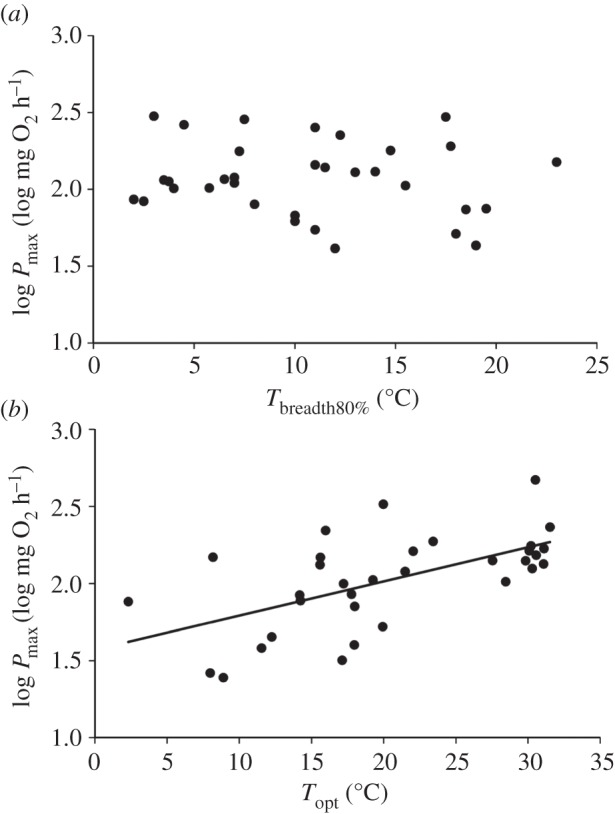
(*a*) Relationship between log-transformed peak aerobic scope (*P*_max_) and thermal performance breadth (*T*_breadth_). For visual representation, data were standardized for body mass and *T*_opt_ using residuals from a PGLS multiple regression of log *P*_max_ versus log body mass and *T*_opt_ (log *P*_max_ = 1.0013(log mass) + 0.0285(*T*_opt_) − 0.9778; *p* < 0.001, *r*^2^ = 0.924), added to the fitted model value for body mass = 300 g and *T*_opt_ = 20°C (the mean body mass and *T*_opt_ for species used in this study, respectively). (*b*) Relationship between *P*_max_ and optimum temperature (*T*_opt_). For this panel, *P*_max_ was standardized to a body mass of 300 g using residuals of a PGLS linear regression of log *P*_max_ versus log body mass (log *P*_max_ = 0.9409(log mass) − 0.3227; *p* < 0.001; *r*^2^ = 0.864). In both panels, each point represents one species (*n* = 28).

PGLS analysis revealed no relationship between *P*_max_ and *T*_breadth_, regardless of the threshold used to define *T*_breadth_ (figure 2*a*, model details for 80% *T*_breadth_ threshold in table 1; for 60%, *p* = 0.188; 70%, *p* = 0.368; 90%, *p* = 0.200; electronic supplementary material, tables S3–S5). *P*_max_ increased with *T*_opt_ (figure 2*b*; PGLS, effect of *T*_opt_, *t* = 4.240, *p* < 0.001). Explanatory variables explained 92.2% of variation in *P*_max_ (*R*^2^). Trends were identical when PGLS models were performed using studies with acclimated animals only (electronic supplementary material, table S2, PGLS, effect of *T*_breadth80%_, *t* = −0.752, *p* = 0.466).

**Table 1.** Summary of the PGLS model testing for the effects of aerobic scope breadth (*T*_breadth80%_,°C), optimal temperature (*T*_opt_, °C) and body mass (log g) on *P*_max_ (log mg O_2_ h^−1^). *r*^2^ = 0.937, *F*_5,21_ = 62.59, *p* < 0.001, *n* = 28 species, *λ* = 0.70. For lifestyle categorization, the reference category is ‘benthic’.

term	estimate	s.e.m.	*t*	*p*
intercept	−1.000	0.252	−3.971	<0.001
*T*_breadth80%_	−0.01	0.008	−1.249	0.226
*T*_opt_	0.03	0.007	4.240	<0.001
log mass	1.01	0.066	15.387	<0.001
lifestyle				
benthopelagic	0.072	0.110	0.660	0.516
pelagic	0.246	0.117	2.095	0.049

## 4. Discussion

We found no evidence of a trade-off between *T*_breadth_ and *P*_max_ for AS across teleost fishes. Evolutionary and plastic changes to peak performance for AS may not necessarily lead to reduced performance over a broader range of temperatures and so there may not be differentiation along a thermal specialist/generalist continuum with respect to AS in teleost fishes. Investment in factors such as gill surface area, heart pumping capacity, tissue vascularization and mitochondrial density should all act to increase *P*_max_ [17,22]. The results here suggest that these features can also increase AS at other temperatures, particularly if compensatory mechanisms allow for plasticity in each in response to temperature (e.g. change in the concentration or isoforms of aerobic enzymes [23]). It has been proposed that AS may constrain geographical distributions of aquatic ectothermic species in the face of a warming climate and other aspects of environmental changes [18]. The findings here suggest that the evolutionary potential of *P*_max_ is not constrained by prior adaptation for a wider *T*_breadth_, or vice versa, depending on the degree of genetic correlation between *P*_max_ and *T*_breadth_. For example, stenothermal species, which may have experienced relaxed selection for *T*_breadth_, may be able to readily evolve a broader performance breadth or shift *P*_max_ in response to changing thermal regimes, at least for AS. There are also other factors influencing plastic and evolutionary responses to thermal regimes that perhaps override or obscure links between *P*_max_ and *T*_breadth_ for AS. For example, a decreased *T*_breadth_ for AS may not compromise fitness to the same extent as a reduced ability to adjust cellular membrane fluidity in response to thermal variation [24]. This could preclude a negative correlation between *P*_max_ and *T*_breadth_ for AS from arising across species through evolutionary processes. It should also be noted that there appears to be several fish species that may not show a decline in AS with increasing temperature before reaching *CT*_max_ [13]_._ In such species, *P*_max_ must also not be constrained by performance breadth for AS.

Our phylogenetically informed analysis spans a variety of species with varying lifestyles. Still, complex interactions among thermal history, body size, lifestyle and evolutionary history could also mask a trade-off between *P*_max_ and *T*_breadth_ for AS. Across fishes, AS differs among benthic, benthopelagic and pelagic species [17], and so it is also possible that selection on AS to support locomotory capacity may outweigh thermal effects on *P*_max_. This interpretation is supported by the observation that pelagic species had a higher *P*_max_ in this study, as compared to benthic and benthopelagic species. (table 1). It must also be considered that species may differ in the percentage of *P*_max_ most relevant for defining *T*_breadth_ [12]. For example, species that perform long migrations might require a higher proportion of *T*_breadth_ to maximize lifetime fitness when compared with more sedentary species.

*P*_max_ was higher in species with a higher *T*_opt_, providing support for the ‘hotter is better’ model [25]. AS in fishes generally increases with temperature until *T*_opt_ and then decreases as temperature increases further (though see [13]). It is unlikely that species would live in habitats with temperatures much higher than *T*_opt_, given that thermal performance curves can be asymmetrical, with performance dropping more steeply above *T*_opt_ [12]. To date, most studies have not measured AS at sufficient temperature points to permit complex asymmetrical modelling [19] and so we have little understanding of how curve asymmetry may be linked with thermal specialization for AS. This may be especially relevant for the many tropical fishes that have a decreased thermal window between *CT*_min_ and *CT*_max_ [26]. Future studies examining changes in AS with temperature in ectotherms should perform measurements across as many temperatures as possible to permit complex modelling of asymmetrical performance curves.

## Supplementary Material

Table S1

## Supplementary Material

Figure S1

## Supplementary Material

Figure S2

## Supplementary Material

Table S2

## Supplementary Material

Table S3-S5

## Data accessibility

The data from this study are available as electronic supplementary material.
