# Supplementary material for: Is there a trade-off between peak performance and performance breadth across temperatures for aerobic scope in teleost fishes?
Source: Biol Lett. 2016 Sep;12(9):20160191. doi: 10.1098/rsbl.2016.0191 (PMC5046912; doi:10.1098/rsbl.2016.0191)
Supplement: Figure S1 [file rsbl20160191supp2.docx]

**Figure S1.** A) Relationship between model-derived P_max_ and maximum aerobic scope (AS) measured in the original study from which data were extracted (P_max_ = 0.998(AS) + 3.876; p < 0.001, r^2^ = 0.996). B) Relationship between model-derived T_opt_ and the maximum temperature (T) at which AS was measured in the original study from which the data were extracted (T_opt_ = 0.962(T) + 0.415; p < 0.001, r^2^ = 0.973). In both panels, each point represents one study and species (n = 28).
