# Supplementary material for: Is there a trade-off between peak performance and performance breadth across temperatures for aerobic scope in teleost fishes?
Source: Biol Lett. 2016 Sep;12(9):20160191. doi: 10.1098/rsbl.2016.0191 (PMC5046912; doi:10.1098/rsbl.2016.0191)
Supplement: Figure S2 [file rsbl20160191supp3.docx]

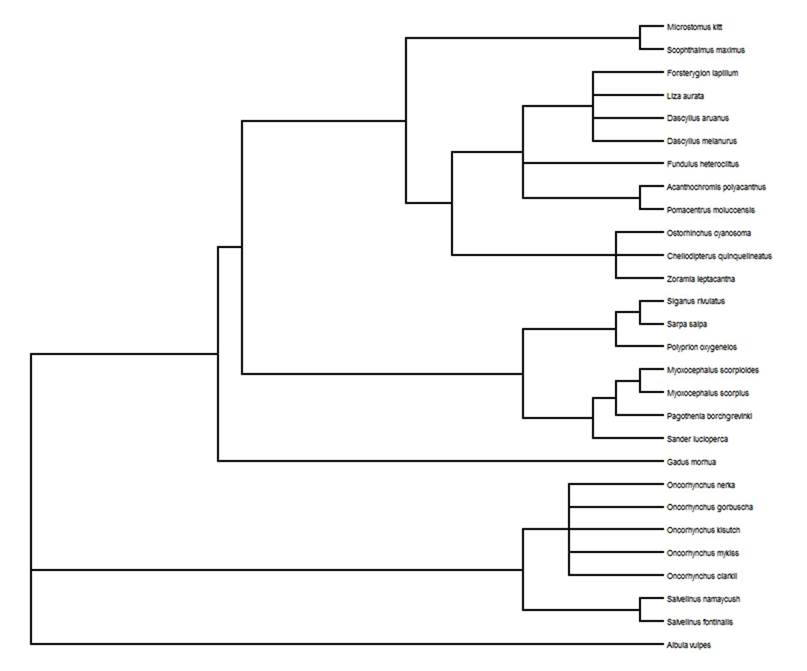
**Figure S2.** Data were analysed using the phylogenetic generalised least squares (PGLS) method [[1](#_ENREF_1), [2](#_ENREF_2)] with the ‘caper’ package [[3](#_ENREF_3)] in R, applying a phylogeny generated from the comprehensive tree of life [[4](#_ENREF_4)] using the ‘rotl’ package [[5](#_ENREF_5)]. The branch lengths in the tree were estimated using Grafen’s arbitrary branch lengths transformation [[2](#_ENREF_2)] (branch lengths set to a length equal to the number of descendant tips minus one). A measure of phylogenetic correlation, λ [[6](#_ENREF_6)] was estimated by ﬁtting PGLS models with different values of λ and finding the value that maximises the log likelihood. Thus the degree to which trait evolution deviates from Brownian motion (λ = 1) was determined by modifying the covariance matrix using the maximum-likelihood value of λ, which is a multiplier of the off-diagonal elements of the covariance matrix (i.e. those quantifying the degree of relatedness between species).

**Literature Cited**

[1] Garland Jr, T. & Ives, A.R. 2000 Using the past to predict the present: confidence intervals for regression equations in phylogenetic comparative methods. *The American Naturalist* **155**, 346-364.

[2] Grafen, A. 1989 The phylogenetic regression. *Philosophical Transactions of the Royal Society of London. Series B, Biological Sciences* **326**, 119-157.

[3] Orme, D. 2013 The caper package: comparative analysis of phylogenetics and evolution in R. *R package version* **5**.

[4] Hinchliff, C.E., Smith, S.A., Allman, J.F., Burleigh, J.G., Chaudhary, R., Coghill, L.M., Crandall, K.A., Deng, J., Drew, B.T. & Gazis, R. 2015 Synthesis of phylogeny and taxonomy into a comprehensive tree of life. *Proceedings of the National Academy of Sciences* **112**, 12764-12769.

[5] Michonneau, F., Brown, J.W. & Winter, D. 2015 rotl, an R package to interact with the Open Tree of Life data. (PeerJ PrePrints.

[6] Freckleton, R.P., Harvey, P.H. & Pagel, M. 2002 Phylogenetic analysis and comparative data: a test and review of evidence. *American Naturalist* **160**, 712-726.
