# Supplementary material for: Is there a trade-off between peak performance and performance breadth across temperatures for aerobic scope in teleost fishes?
Source: Biol Lett. 2016 Sep;12(9):20160191. doi: 10.1098/rsbl.2016.0191 (PMC5046912; doi:10.1098/rsbl.2016.0191)
Supplement: Table S2 [file rsbl20160191supp4.docx]

| term | estimate | s.e.m | | *t* | | *p* | | |  |  |
| --- | --- | --- | --- | --- | --- | --- | --- | --- | --- | --- |
| intercept  T_breadth_ | -0.795  -0.006 | | 0.268  0.008 | | -2.964  -0.752 | | 0.011  0.466 |  | | |
| T_opt_ | 0.027 | | 0.008 | | 3.35 | | 0.005 |  | |  |
| log mass | 0.923 | | 0.084 | | 10.956 | | <0.001 |  | |  |
| lifestyle benthopelagic | 0.021 | | 0.103 | | 0.204 | | 0.841 |  | |  |
| pelagic | 0.155 | | 0.17 | | 0.91 | | 0.379 |  | |  |

**Table S2.** Summary of the phylogenetic least squares regression model testing for the effects of thermal performance breadth for aerobic scope (80 % of P_max_), optimal temperature (T_opt_), body mass (log g) and lifestyle (benthic, benthopelagic, or pelagic) on maximum aerobic scope (P_max_; log mg O_2_ h^-1^). For lifestyle categorisation, the reference category is ‘benthic’. This analysis only used studies in which fish were acclimated to each experimental temperature. r^2^ = 0.94, F_5, 13_= 40.76, p<0.001, n= 20 species, λ=0.659.
