# Supplementary material for: Is there a trade-off between peak performance and performance breadth across temperatures for aerobic scope in teleost fishes?
Source: Biol Lett. 2016 Sep;12(9):20160191. doi: 10.1098/rsbl.2016.0191 (PMC5046912; doi:10.1098/rsbl.2016.0191)
Supplement: Table S3-S5 [file rsbl20160191supp5.docx]

| term | estimate | s.e.m | | *t* | | *p* | | |  |  |
| --- | --- | --- | --- | --- | --- | --- | --- | --- | --- | --- |
| intercept  T_breadth_ | -0.856  -0.007 | | 0.299  0.005 | | -2.862  -1.362 | | 0.009  0.188 |  | | |
| T_opt_ | 0.025 | | 0.007 | | 3.533 | | 0.002 |  | |  |
| log mass | 0.975 | | 0.065 | | 15.021 | | <0.001 |  | |  |
| lifestyle benthopelagic | 0.088 | | 0.081 | | 1.093 | | 0.287 |  | |  |
| pelagic | 0.281 | | 0.105 | | 2.676 | | 0.014 |  | |  |

**Table S3.** Summary of the phylogenetic least squares regression model testing for the effects of thermal performance breadth for aerobic scope (60 % of P_max_), optimal temperature (T_opt_), body mass (log g) and lifestyle (benthic, benthopelagic, or pelagic) on maximum aerobic scope (P_max_; log mg O_2_ h^-1^). r^2^ = 0.93, F_5, 21_= 55.19, p<0.001, n= 28 species, λ=1. For lifestyle categorisation, the reference category is ‘benthic’.

| term | estimate | s.e.m | | *t* | | *p* | | |  |  |
| --- | --- | --- | --- | --- | --- | --- | --- | --- | --- | --- |
| intercept  T_breadth_ | -1.01  -0.006 | | 0.255  0.006 | | -3.955  -0.919 | | <0.001  0.368 |  | | |
| T_opt_ | 0.03 | | 0.007 | | 4.147 | | <0.001 |  | |  |
| log mass | 1.005 | | 0.066 | | 15.177 | | <0.001 |  | |  |
| lifestyle benthopelagic | 0.067 | | 0.112 | | 0.596 | | 0.558 |  | |  |
| pelagic | 0.248 | | 0.120 | | 2.065 | | 0.052 |  | |  |

**Table S4.** Summary of the phylogenetic least squares regression model testing for the effects of thermal performance breadth for aerobic scope (70 % of P_max_), optimal temperature (T_opt_), body mass (log g) and lifestyle (benthic, benthopelagic, or pelagic) on maximum aerobic scope (P_max_; log mg O_2_ h^-1^). r^2^ = 0.935, F_5, 21_= 60.86, p<0.001, n= 28 species, λ=0.684. For lifestyle categorisation, the reference category is ‘benthic’.

**Table S5.** Summary of the phylogenetic least squares regression model testing for the effects of thermal performance breadth for aerobic scope (90 % of P_max_), optimal temperature (T_opt_), body mass (log g) and lifestyle (benthic, benthopelagic, or pelagic) on maximum aerobic scope (P_max_; log mg O_2_ h^-1^). r^2^ = 0.929, F_5, 21_= 54.92, p<0.001, n= 28 species, λ=1. For lifestyle categorisation, the reference category is ‘benthic’.

| term | estimate | s.e.m | | *t* | | *p* | | |  |  |
| --- | --- | --- | --- | --- | --- | --- | --- | --- | --- | --- |
| intercept  T_breadth_ | -0.858  -0.015 | | 0.3  0.011 | | -2.864  -1.324 | | 0.009  0.2 |  | | |
| T_opt_ | 0.025 | | 0.007 | | 3.509 | | 0.002 |  | |  |
| log mass | 0.975 | | 0.065 | | 14.98 | | <0.001 |  | |  |
| lifestyle benthopelagic | 0.087 | | 0.081 | | 1.078 | | 0.293 |  | |  |
| pelagic | 0.281 | | 0.105 | | 2.673 | | 0.014 |  | |  |
